# Notes from Professor Kent’s Lecture upon Arum Triphyllum

**Published:** 1885-12

**Authors:** 


### NOTES FROM PROFESSOR KENT'S LECTURE UPON ARUM TRIPHYLLUM.

This remedy is occasionally indicated in typhus, intermittent with typhoid symptoms, and the febricula of little children by peculiar and distinct symptoms. Little children *bore their fingers into their nose.* There is more or less pain, still it bores and picks its nose—even tries to get the entire fist in. These symptoms must be accompanied by scanty or suppressed urine. There is more or less delirium, general redness of the eyes, catarrhal state of the nose, with corrosive discharge, which causes itching and tingling, provoking the patient to bore. Coryza fluid and acrid; water runs down over the lip and produces a raw streak in its course; excoriates the upper lip and wings of the nose. During day yellow, thick mucous discharge; nose stopped up, worse left side; must breathe through the mouth; worse in the morning; drink passes up through the nose. Corrosive nasal discharge generally, but sometimes thin and bland. (Ars. has a corrosive discharge but it does not compel the patient to pick the nose.) The important feature is, the child will pick and bore in the nose continually. Picking of the lips until they bleed. The corners of the mouth are sore. This remedy is sometimes indicated in scarlatina. (Bell. Arum-t. patients desire to be covered, Apis uncovered.) Swelling of the submaxillary glands. (Merc. if submaxillary glands are alone swollen.) Excessive salivation. Buccal cavity sore, raw, and bleeding. The first indication of the action of this remedy is that the urine begins to flow. In all of these acute inflammatory states you must repeat your remedy every hour. Clergymen or singers have sore throat or laryngitis, brought about by excessive speaking or singing. Give them Arum-t. This is not provoked by a cold usually, but immoderate use producing a weakness of the vocal cords.

